# *BRCA* Genetic Testing and Receipt of Preventive Interventions Among Women Aged 18–64 Years with Employer-Sponsored Health Insurance in Nonmetropolitan and Metropolitan Areas — United States, 2009–2014

**DOI:** 10.15585/mmwr.ss6615a1

**Published:** 2017-09-08

**Authors:** Katherine Kolor, Zhuo Chen, Scott D. Grosse, Juan L. Rodriguez, Ridgely Fisk Green, W. David Dotson, M. Scott Bowen, Julie A. Lynch, Muin J. Khoury

**Affiliations:** 1 Office of Public Health Genomics, Division of Public Health Information Dissemination, Center for Surveillance, Epidemiology, and Laboratory Services, CDC, Atlanta, Georgia; 2 CFO Leasing, Inc., Atlanta, Georgia; 3 Department of Health Policy and Management, College of Public Health, University of Georgia, Athens, Georgia; 4 National Center on Birth Defects and Developmental Disabilities, CDC, Atlanta, Georgia; 5 Division of Cancer Prevention and Control, National Center for Chronic Disease Prevention and Health Promotion, CDC, Atlanta, Georgia; 6 Carter Consulting, Inc., Atlanta, Georgia; 7 US Department of Veterans Affairs Salt Lake City Healthcare System, Salt Lake City, Utah

## Abstract

**Problem/Condition:**

Genetic testing for breast cancer 1 (*BRCA1*) and breast cancer 2 (*BRCA2*) gene mutations can identify women at increased risk for breast and ovarian cancer. These testing results can be used to select preventive interventions and guide treatment. Differences between nonmetropolitan and metropolitan populations in rates of *BRCA* testing and receipt of preventive interventions after testing have not previously been examined.

**Period Covered:**

2009–2014.

**Description of System:**

Medical claims data from Truven Health Analytics MarketScan Commercial Claims and Encounters databases were used to estimate rates of *BRCA* testing and receipt of preventive interventions after *BRCA* testing among women aged 18–64 years with employer-sponsored health insurance in metropolitan and nonmetropolitan areas of the United States, both nationally and regionally.

**Results:**

From 2009 to 2014, *BRCA* testing rates per 100,000 women aged 18–64 years with employer-sponsored health insurance increased 2.3 times (102.7 to 237.8) in metropolitan areas and 3.0 times (64.8 to 191.3) in nonmetropolitan areas. The relative difference in *BRCA* testing rates between metropolitan and nonmetropolitan areas decreased from 37% in 2009 (102.7 versus 64.8) to 20% in 2014 (237.8 versus 191.3). The relative difference in *BRCA* testing rates between metropolitan and nonmetropolitan areas decreased more over time in younger women than in older women and decreased in all regions except the West. Receipt of preventive services 90 days after *BRCA* testing in metropolitan versus nonmetropolitan areas throughout the period varied by service: the percentage of women who received a mastectomy was similar, the percentage of women who received magnetic resonance imaging of the breast was lower in nonmetropolitan areas (as low as 5.8% in 2014 to as high as 8.2% in 2011) than metropolitan areas (as low as 7.3% in 2014 to as high as 10.3% in 2011), and the percentage of women who received mammography was lower in nonmetropolitan areas in earlier years but was similar in later years.

**Interpretation:**

Possible explanations for the 47% decrease in the relative difference in *BRCA* testing rates over the study period include increased access to genetic services in nonmetropolitan areas and increased demand nationally as a result of publicity. The relative differences in metropolitan and nonmetropolitan *BRCA* testing rates were smaller among women at younger ages compared with older ages.

**Public Health Action:**

Improved data sources and surveillance tools are needed to gather comprehensive data on *BRCA* testing in the United States, monitor adherence to evidence-based guidelines for *BRCA* testing, and assess receipt of preventive interventions for women with *BRCA* mutations. Programs can build on the recent decrease in geographic disparities in receipt of *BRCA* testing while simultaneously educating the public and health care providers about U.S. Preventive Services Task Force recommendations and other clinical guidelines for *BRCA* testing and counseling.

## Introduction

Genetic mutations in the breast cancer 1 (*BRCA1*) and breast cancer 2 (*BRCA2*) genes can increase breast cancer risk in women by age 70 years from 7% to an estimated 45%–65% and can increase ovarian cancer risk by age 70 years from 0.6% to 17%–39% (*1*,*2*). Genetic testing based on personal and family health history criteria can identify women with *BRCA* mutations who could benefit from preventive interventions that can decrease cancer risk. *BRCA* testing for women who have received these cancer diagnoses can be used to make surgical and drug treatment decisions (*3*,*4*). 

Interventions for women with *BRCA* mutations for cancer prevention and earlier detection include enhanced screening (e.g., receiving mammograms at a younger age and more frequently than lower risk women along with magnetic resonance imaging [MRI]); risk-reducing medications such as tamoxifen or raloxifene; and prophylactic surgery to remove the breasts, ovaries, or both. Prophylactic mastectomy can reduce the risk for breast cancer by 85%–100%, and prophylactic oophorectomy can reduce the risk for ovarian cancer by 69%–100% and breast cancer by 37%–100% (*1*). For women who have breast cancer, prophylactic contralateral mastectomy might be offered as an option to reduce the risk for contralateral breast cancers (*5*). *BRCA* testing is approved by the Food and Drug Administration as a companion diagnostic tool to guide treatment with poly(ADP-ribose) polymerase (PARP) inhibitors among women with ovarian cancer who have already undergone multiple lines of chemotherapy (*6*,*7*). Phase III clinical trials have reported promising results for the PARP inhibitor olaparib as a treatment for breast cancer in women with *BRCA* mutations (*8*). 

Several clinical guidelines are available to help specialty and primary care providers to determine whether *BRCA* genetic counseling and testing is appropriate for their patients. The 2013 U.S. Preventive Services Task Force (USPSTF) *BRCA* guideline recommends that primary care providers assess women for increased risk for *BRCA* mutations using family health history to identify women who might benefit from genetic counseling (*1*). The National Comprehensive Cancer Network (NCCN) guidelines specify family and personal history criteria that warrant additional genetic risk assessments (*3*). In addition, the USPSTF and NCCN guidelines review preventive interventions for women identified with *BRCA* mutations. When a *BRCA* mutation is identified, other family members can be tested for the known mutation (i.e., cascade testing) to identify others at increased risk for cancer (*1*,*3*). A *Healthy People 2020* objective is to increase rates of genetic counseling for women with a family health history indicating an increased risk for breast or ovarian cancers (*9*). *BRCA* genetic counseling and testing are usually covered, in network without cost sharing, by most health insurance plans as preventive services when used in accordance with USPSTF recommendations (*10*).

*BRCA* testing guidelines generally recommend both pretest and posttest genetic counseling by a trained health care provider (*1*,*11*). As of 2016, approximately 5,000 genetic counselors were practicing in the United States (*12*). The geographic distribution of genetic service providers has historically been uneven, with a concentration in urban areas and academic medical centers. However, a 2016 survey of genetic counselors found that nearly half reported serving smaller, more rural populations (*12*). Alternative genetic services delivery models, such as telephone counseling (*13*), outreach clinic services (*14*), and telemedicine genetic services (*15*–*17*), are being incorporated by genetic counselors to complement in-person counseling and to help improve overall access to care (e.g., in rural areas). In addition, genetic services, such as *BRCA* genetic counseling and testing, are being offered by specialists who are not geneticists, including oncologists and obstetrician-gynecologists (*18*,*19*).

A comparison of changes over time in *BRCA* testing rates and receipt of preventive interventions after *BRCA* testing in urban and rural areas at the national and regional levels could help to document the evolution of differences in service use. However, the available data for monitoring *BRCA* testing in the U.S. population and its subgroups are limited. For example, claims data can be used to assess use of health services, and specific billing codes for *BRCA* testing have been available since 2001 (*20*). However, *BRCA* test billing codes have changed over time, and before 2013, nonspecific billing codes were also used for *BRCA* testing, which hinders complete ascertainment of these tests from claims data (*21*). Furthermore, numerous factors might influence variations in *BRCA* test use in the United States. For example, a substantial increase in *BRCA* testing occurred in 2013 after publication of a celebrity’s editorial describing her decision to receive a preventive double mastectomy after *BRCA* testing indicated that she carried a *BRCA* gene mutation (*22*,*23*). In addition, physicians reported conducting more testing after a direct-to-consumer advertising campaign that was intended to raise awareness about *BRCA* testing among women with a personal or family history of breast or ovarian cancer (*24*). Racial/ethnic disparities in rates of *BRCA* testing and follow-up preventive services have been reported (*25*), attributed in part to socioeconomic factors and physician referral patterns (*26*).

To examine differences over time and by census region in *BRCA* testing rates in nonmetropolitan and metropolitan areas in the United States, this report analyzes health claims data from the Truven Health Analytics MarketScan Commercial Claims and Encounters (CCAE) databases. This report presents rates of *BRCA* testing (for any reason other than testing for known Ashkenazi mutations) from 2009 to 2014 among women aged 18–64 years. In addition, this report describes receipt of preventive services (i.e., mammography, breast MRI, and mastectomy) and genetic counseling among women who had *BRCA* tests. These findings can be used by public health officials and practitioners to better understand differences in the implementation of genomic medicine applications in metropolitan and nonmetropolitan areas and identify areas of potential need and growth.

## Methods

Medical claims data from 2009 to 2014 for women aged 18–64 years were extracted from Truven Health Analytics MarketScan CCAE databases (*27*), a proprietary data system integrating information from inpatient services, outpatient services (including laboratory tests), and outpatient pharmacies provided by a nationwide convenience sample of employers and employer-sponsored commercial insurance plans that cover employees and their dependents. The MarketScan CCAE databases contain enrollment data and longitudinally linked health care use and expenditure data. MarketScan claims and patient-level information, including age, sex, geographic location, diagnostic codes, and procedure codes, were analyzed. Both partial-year and full-year enrollees were included. Residential status (nonmetropolitan and metropolitan) of enrollees was mapped from the five-digit zip code of the primary beneficiary by Truven Health Analytics based on the Office of Management and Budget delineations for metropolitan statistical areas (MSAs) available through the U.S. census website (*27*). The residential status of women who changed their status (i.e., from nonmetropolitan to metropolitan or the reverse) was determined by where they lived the most months during that year.

Receipt of both preventive-associated and treatment-associated *BRCA* testing was ascertained from outpatient and inpatient claims. Claims of *BRCA* testing were extracted using Healthcare Common Procedure Coding System (HCPCS) codes (S3818–S3823) and Current Procedural Terminology (CPT) codes (81211–81217). Four subtypes of *BRCA* tests were defined based on billing codes: full-gene sequencing, known mutation tests, Ashkenazi panel, and large rearrangement tests (which are typically done at the same time as or after full-gene sequencing tests). Women who had *BRCA* panel tests for Ashkenazi mutations were excluded from the final analyses of *BRCA* testing rates to avoid inflating differences between metropolitan and nonmetropolitan areas because almost all Ashkenazi panel testing was concentrated in metropolitan areas. Women who had Ashkenazi panel testing accounted for approximately 5% of women who had any *BRCA* testing during the study period and ≤0.1% of all women aged 18–64 years. When calculating *BRCA* testing rates, women who had Ashkenazi panel tests were excluded from the numerator.  However, the data contain no measure of ethnicity that could be used to exclude women of Ashkenazi ancestry from the denominator. 

The annual calendar-year receipt rates of any *BRCA* test and subtypes were calculated as follows: the number of women enrollees aged 18–64 years who had at least one claim in the calendar year that contained one of the *BRCA* testing procedure codes divided by the total number of women aged 18–64 years enrolled at any point during that year, multiplied by 100,000. For each study year, enrollee age was defined as the age on January 1, or at the start of enrollment period for partial-year enrollees. Rates also were calculated by age group (18–34, 35–44, 45–54, and 55–64 years) and by U.S. census region (Northeast, Midwest, South, and West) (*28*). Adjusting *BRCA* testing rates in metropolitan and nonmetropolitan areas by age and region using 2010 U.S. census data did not affect the general findings or conclusions. Therefore, only unadjusted *BRCA* testing rates are reported.

Receipt of preventive services within 90 days or 1 year after *BRCA* testing was examined for mastectomy (CPT codes 19303 and 19304, and *International Classification of Diseases,* *Ninth Revision, Clinical Modification* ([ICD-9-CM] procedure codes 85.33–85.36 and 85.41–85.44), breast MRI (CPT code 77059) and screening and diagnostic mammography (HCPCS codes G0202, G0204, and G0206 and CPT codes 77055, 77056, and 77057). The CPT code for MRI does not distinguish between screening and diagnostic indications. Both screening and diagnostic codes were used for mammography because certain physicians might bill for screening mammograms using diagnostic codes (*29*). Receipt of formal genetic counseling was examined within 90 days before testing and within 90 days after *BRCA* testing to ensure inclusion of pretest and posttest counseling (HCPCS code S0265 and CPT code 96040).

Chi-square tests were used for statistical comparisons, with no corrections for multiple comparisons. The relative difference in *BRCA* testing rates between metropolitan and nonmetropolitan areas was calculated as the difference between the metropolitan and nonmetropolitan rates as a proportion of the metropolitan rate. Differences for rates and relative differences were considered significant at p<0.05. 

## Results

### MarketScan CCAE Sample Characteristics

From 2009 to 2014, the number of women aged 18–64 years included annually in the MarketScan CCAE databases ranged from as low as 15.5 million (in 2009) to as high as 20.6 million (in 2012) (Supplementary Table, https://stacks.cdc.gov/view/cdc/47271). The proportion of women living in nonmetropolitan areas decreased over time from 15.3% in 2009 to 12.9% in 2014. Women in the MarketScan CCAE databases were older in nonmetropolitan areas than in metropolitan areas (Supplementary Table, https://stacks.cdc.gov/view/cdc/47271).

From 2009 to 2014, a total of 164,837 unique enrollees aged 18–64 years were identified in the MarketScan sample as having had *BRCA* tests, with 166,069 person-years of testing (Table 1); 1,221 enrollees had *BRCA* testing in ≥2 years, including 11 with testing in >2 years. Of the 1,221 enrollees who had *BRCA* testing in multiple years, 1,123 (92.0%) lived in metropolitan areas, and 86 (7.0%) lived in nonmetropolitan areas. Twelve (<1.0%) changed residential status. A total of 10.4% of enrollees who had *BRCA* tests during the study period lived in nonmetropolitan areas (n = 17,112) (Table 1).

**TABLE 1:** Number and rate* of *BRCA* testing among women aged 18–64 years^†^ who were enrolled in employer-sponsored health insurance, in metropolitan and nonmetropolitan areas — United States, 2009–2014

Age group and region	2009		2010		2011		2012		2013		2014		Total^§^		
	Metro	Nonmetro	Metro	Nonmetro	Metro	Nonmetro	Metro	Nonmetro	Metro	Nonmetro	Metro	Nonmetro	Metro	Nonmetro	Total
	No. (Rate)	No. (Rate)	No. (Rate)	No. (Rate)	No. (Rate)	No. (Rate)	No. (Rate)	No. (Rate)	No. (Rate)	No. (Rate)	No. (Rate)	No. (Rate)	No. (Rate)	No. (Rate)	No. (Rate)
**Total^¶^**	**13,475 (102.7)**	**1,533 (64.8)**	**16,826 (113.2)**	**1,977 (77.3)**	**21,558 (125.2)**	**2,576 (88.8)**	**25,256 (143.4)**	**2,945 (100.1)**	**33,193 (222.6)**	**3,560 (160.4)**	**38,560 (237.8)**	**4,610 (191.3)**	**147,725 (380.3)**	**17,112 (255.2)**	**164,837 (361.9)**
**Age group (yrs)**															
18–34	1,788 (41.2)	173 (24.3)	2,106 (43.0)	255 (33.7)	3,002 (51.4)	347 (39.0)	3,875 (63.3)	424 (45.0)	5,010 (96.4)	579 (81.5)	6,328 (110.8)	742 (96.6)	**22,030 (142.1)**	**2,511 (103.4)**	**24,541 (136.1)**
35–44	4,059 (137.9)	425 (85.8)	4,929 (147.9)	562 (104.4)	6,175 (166.0)	645 (110.5)	7,275 (193.3)	782 (134.3)	9,384 (297.4)	923 (211.5)	10,818 (318.7)	1,205 (255.8)	**42,335 (515.9)**	**4,519 (337.0)**	**46,854 (490.8)**
45–54	4,918 (151.1)	572 (92.2)	6,251 (168.8)	740 (109.7)	7,696 (184.8)	951 (129.1)	8,736 (209.1)	1,048 (144.1)	11,256 (322.9)	1,229 (228.7)	12,377 (332.5)	1,487 (258.1)	**50,794 (597.1)**	**5,999 (380.6)**	**56,793 (563.3)**
55–64	2,710 (105.2)	363 (67.3)	3,540 (120.9)	420 (71.7)	4,685 (134.3)	633 (91.5)	5,370 (151.3)	691 (99.9)	7,543 (245.9)	829 (154.9)	9,037 (267.0)	1,176 (197.8)	**32,566 (491.0)**	**4,083 (300.1)**	**36,649 (458.5)**
Region**															
Northeast	2,668 (148.6)	89 (83.0)	3,695 (153.7)	227 (104.6)	5,021 (153.0)	482 (120.8)	5,678 (166.9)	471 (117.3)	8,843 (291.3)	471 (195.5)	9,747 (263.6)	750 (206.0)	**35,317 (487.8)**	**2,468 (326.9)**	**37,785 (472.6)**
South	6,114 (101.4)	738 (62.7)	6,373 (112.1)	967 (73.8)	7,205 (119.5)	1,072 (78.4)	8,696 (142.2)	1,278 (96.5)	10,368 (211.9)	1,723 (168.7)	13,653 (238.3)	2,358 (211.9)	**52,071 (348.8)**	**8,103 (256.7)**	**60,174 (332.7)**
Midwest	2,911 (90.5)	517 (65.1)	3,821 (109.5)	524 (74.1)	4,679 (121.3)	716 (90.0)	5,314 (137.9)	850 (105.0)	5,741 (195.8)	939 (149.9)	6,439 (216.1)	1,042 (159.7)	**28,717 (343.7)**	**4,561 (236.9)**	**33,278 (323.7)**
West	1,753 (85.9)	189 (68.6)	2,876 (89.3)	257 (80.5)	3,853 (112.2)	306 (90.7)	4,907 (130.1)	346 (84.9)	7,312 (204.6)	426 (128.9)	7,865 (233.0)	460 (164.7)	**28,319 (367.3)**	**1,976 (232.2)**	**30,295 (353.9)**

**Source:** Truven Health Analytics. Truven Health MarketScan research databases, commercial claims and encounters Medicare supplemental, data year 2009–2014. Ann Arbor, MI: Truven Health Analytics. http://truvenhealth.com/markets/life-sciences/products/data-tools/marketscan-databases

**Abbreviations:** *BRCA* = breast cancer (gene); metro = metropolitan; nonmetro = nonmetropolitan.

* Per 100,000 population of women aged 18-64 years who were enrolled in employer-sponsored health insurance in the MarketScan Commercial Claims and Encounters database. Pairwise comparison of *BRCA* testing rates in metropolitan versus nonmetropolitan areas were significantly different for each year and total across study years (chi-square test, p<0.05).

^†^ Women who had *BRCA* testing for Ashkenazi mutations were excluded from the numerator but not the denominator.

**^§^** Combined data for total sample of women aged 18–64 years corrected for multiyear enrollment. Data combined for women aged 18–64 years who had *BRCA* tests, 2009–2014, corrected for the <1% of women who had *BRCA* testing in >1 year.

**^¶^** During 2009–2014, the number of women aged 18–64 years included annually in the MarketScan Commercial Claims and Encounters databases ranged from as low as 15.5 million (in 2009) to as high as 20.6 million (in 2012) (Supplementary Table, https://stacks.cdc.gov/view/cdc/47271).

** Region percentages might not add up to 100% because of missing data.

### Annual *BRCA* Testing Rates from 2009 to 2014

**FIGURE 1:**
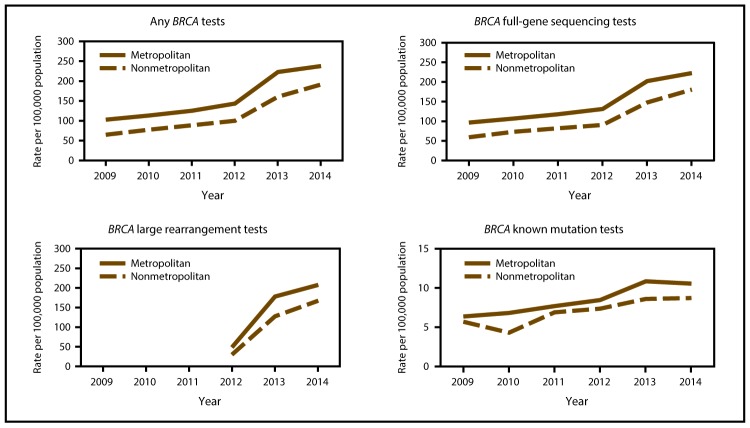
*BRCA* testing rates* among women aged 18–64 years who were enrolled in employer-sponsored health insurance, in metropolitan and nonmetropolitan areas, by test subtype**^†^** — United States, 2009–2014 **Source:** Truven Health Analytics. Truven Health MarketScan research databases, commercial claims and encounters Medicare supplemental, data year 2009–2014. Ann Arbor, MI: Truven Health Analytics. http://truvenhealth.com/markets/life-sciences/products/data-tools/marketscan-databases **Abbreviations:** *BRCA* = breast cancer (gene); CPT = Current Procedural Terminology; HCPCS = Healthcare Common Procedure Coding System. * The denominator is the total number of women aged 18–64 years enrolled in MarketScan Commercial Claims and Encounters databases at any point during that year. ^†^ Three subtypes of *BRCA* tests were defined based on the following billing codes: full-gene sequencing (CPT codes 81211, 81214, and 81216 and HCPCS codes S3818, S3819, and S3820), known mutation tests (CPT codes 81215 and 81217 and HCPCS code S3822), and large rearrangement tests (CPT code 81213). The specific billing code for large rearrangement tests was introduced in 2012.

From 2009 to 2014, *BRCA* testing rates per 100,000 women aged 18–64 years enrolled in employer-sponsored health insurance (MarketScan database) increased 2.3 times (102.7 to 237.8) in metropolitan areas and 3.0 times (64.8 to 191.3) in nonmetropolitan areas (Table 1, Figure 1). The relative difference in testing rates decreased by almost half (47%) from 36.9% in 2009 to 19.6% in 2014. *BRCA* testing rates were higher in metropolitan areas than in nonmetropolitan areas throughout the study period for women having any *BRCA* test and all *BRCA* test subtypes a (Figure 1). In 2014, the proportions of test subtypes were similar in metropolitan and nonmetropolitan areas, with 93.6% (metropolitan) and 94.3% (nonmetropolitan) of women having full-gene sequencing tests, 87.3% (metropolitan) and 87.5% (nonmetropolitan) of women having large rearrangement tests, and 4.4% (metropolitan) and 4.6% (nonmetropolitan) of women having known mutation tests (data not shown).

#### *BRCA* Testing Rates by Age Group

Women who had any *BRCA* test were significantly older on average in nonmetropolitan areas than in metropolitan areas (combined across the study period); however, the youngest women (18–34 years) were similarly represented at 14.7% and 14.9%, respectively (Table 1). For all age groups, *BRCA* testing rates were higher in metropolitan areas than in nonmetropolitan areas across the study period. Women aged 18–34 years had the highest relative increase in *BRCA* testing compared with older age groups both for metropolitan and nonmetropolitan areas. The relative difference in *BRCA* testing rates between metropolitan and nonmetropolitan areas decreased more from 2009 to 2014 in younger women than in older women, and the relative metropolitan-nonmetropolitan differences in *BRCA* testing rates were lower in younger age groups in 2014: 12.8% (18–34 years), 19.7% (35–44 years), 22.4% (45–54 years), and 25.9% (55–64 years).

#### *BRCA* Testing Rates by Region

In each of the four regions, *BRCA* testing rates among women aged 18–64 years increased over the study period both in metropolitan and nonmetropolitan areas (Table 1). *BRCA* testing rates were highest in the Northeast across the study period in both metropolitan and nonmetropolitan areas, except in 2014 when the nonmetropolitan rates in the Northeast and South were comparable. *BRCA* testing rates generally increased more in nonmetropolitan areas than metropolitan areas across the study period in all regions except the West. *BRCA* testing rates showed the greatest relative increase over the study period in the nonmetropolitan South (3.4 times), which accounted for 47% of the nonmetropolitan sample. In the most recent study years (2013–2014), the South had the smallest relative and absolute differences in metropolitan and nonmetropolitan *BRCA* testing rates among women aged 18–64 years (Table 1).

### Receipt of Preventive Interventions and Genetic Counseling Among Women Who Received *BRCA* Testing

**FIGURE 2:**
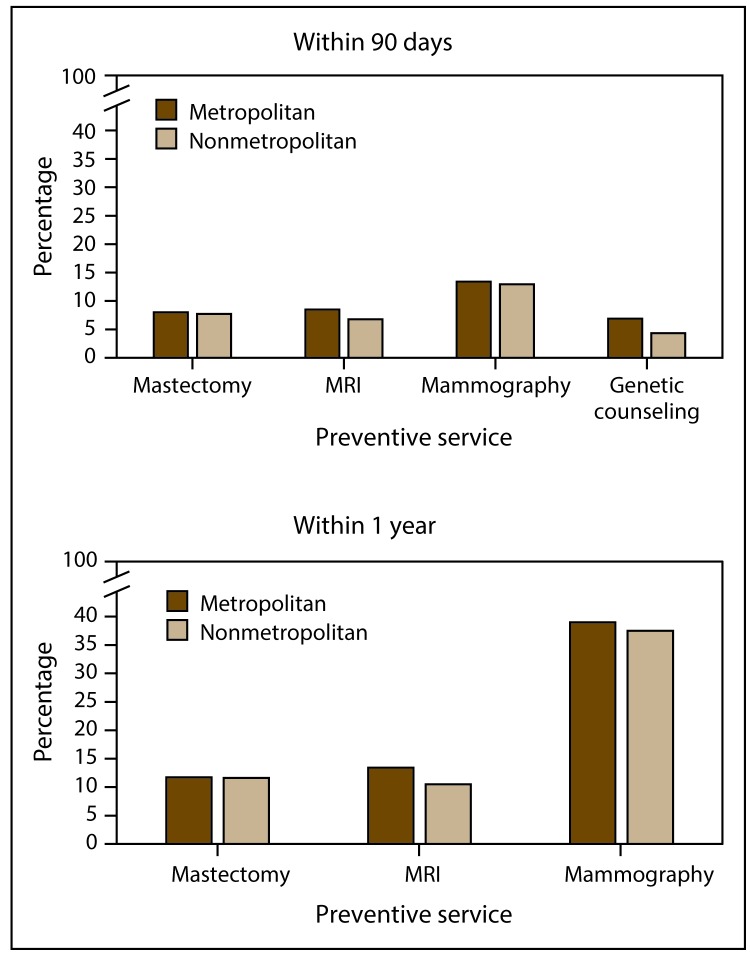
Percentage of women who received preventive services* associated with *BRCA* testing (within 90 days and 1 year of testing**^†^**) among women aged 18–64 years who were enrolled in employer-sponsored health insurance in metropolitan and nonmetropolitan areas**^§^** — United States, 2009–2014 **Source:** Truven Health Analytics. Truven Health MarketScan research databases, commercial claims and encounters Medicare supplemental, data year 2009– 2014. Ann Arbor, MI: Truven Health Analytics. http://truvenhealth.com/markets/life-sciences/products/data-tools/marketscan-databases **Abbreviations:** *BRCA* = breast cancer (gene); CPT = Current Procedural Terminology; HCPCS = Healthcare Common Procedure Coding System; ICD-9-CM = *International Classification of Diseases, Ninth Revision, Clinical Modification;* MRI = magnetic resonance imaging. * Data combined for women aged 18–64 years who had *BRCA* tests, 2009–2014, corrected for the <1% of women who had *BRCA* testing in >1 year. Women who had *BRCA* testing for Ashkenazi mutations were excluded from the analyses.. ^†^ Mastectomy, MRI, and mammography: within 90 days or 1 year after *BRCA* testing; genetic counseling: 90 days before and 90 days after *BRCA* testing. Preventive services were defined based on the following billing codes: mastectomy (CPT codes 19303 and 19304 and ICD-9-CM procedure codes 85.33–85.36 and 85.41–85.44), breast MRI (CPT code 77059), and screening and diagnostic mammography (HCPCS codes G0202, G0204, and G0206 and CPT codes 77055, 77056, and 77057). ^§^ Nonmetropolitan areas had significantly (p<0.05, chi-square test) lower rates of MRI within 90 days or 1 year after testing, genetic counseling within 90 days before and 90 days after testing, and mammography within 1 year after testing.

The percentage of women who received a mastectomy within 90 days or 1 year after *BRCA* testing peaked in 2011 (Table 2). Similar percentages were observed across the study period in metropolitan and nonmetropolitan areas (Table 2). Annual percentages within 90 days after *BRCA* testing ranged from 6.5%–9.6% in metropolitan areas and 5.7%–10.4% in nonmetropolitan areas (Table 2). Annual percentages of mammography within 90 days after *BRCA* testing ranged from 13.0%–14.0% in metropolitan areas and 11.5%–14.1% in nonmetropolitan areas (Table 2). The percentage of women who received a breast MRI within 90 days or 1 year after *BRCA* testing peaked in 2011 and was higher across the study period in metropolitan areas compared with nonmetropolitan areas, ranging from 11.3%–15.8% compared with 8.6%–12.8% in nonmetropolitan areas within 1 year; the difference was not statistically significant for 90-day follow-up in 2010 (Table 2). The percentage of women who received genetic counseling identified through billing codes within 90 days before *BRCA* testing and 90 days after testing was higher in metropolitan areas than in nonmetropolitan areas across the study period; from 2009 to 2014, genetic counseling increased from 5.3% to 8.0% in metropolitan areas and from 3.8% to 5.2% in nonmetropolitan areas (Table 2). When combining data across the study period, nonmetropolitan areas had significantly lower percentages of women who received an MRI within 90 days or 1 year after testing, genetic counseling within 90 days before and 90 days after testing, and mammography within 1 year after testing (Figure 2).

**TABLE 2:** Percentage of women who received preventive services* associated with *BRCA* testing among women aged 18–64 years^†^ enrolled in employer-sponsored health insurance, in metropolitan and nonmetropolitan areas — United States, 2009–2014

Preventive service	2009 (%)				2010 (%)				2011 (%)				2012 (%)				2013 (%)				2014 (%)				Total (%)^§^					
	Metro		Nonmetro		Metro		Nonmetro		Metro		Nonmetro		Metro		Nonmetro		Metro		Nonmetro		Metro		Nonmetro		Metro		Nonmetro		Total	
	N = 13,475		N = 1,533		N = 16,826		N = 1,977		N = 21,558		N = 2,576		N = 25,256		N = 2,945		N = 33,193		N = 3,560		N = 38,560		N = 4,610		N = 147,725		N = 17,112		N = 164,837	
	90 days^¶^	1 yr**	90 days	1 yr	90 days	1 yr	90 days	1 yr	90 days	1 yr	90 days	1 yr	90 days	1 yr	90 days	1 yr	90 days	1 yr	90 days	1 yr	90 days	1 yr	90 days	1 yr	90 days	1 yr	90 days	1 yr	90 days	1 yr
Mastectomy	8.1	11.5	7.8	12.5	9.0	13.2	9.8	13.5	9.6	14.0	10.4	15.3	9.4	13.2	8.8	12.2	7.6	11.1	6.7	10.8	6.5^††^	9.5	5.7^††^	8.6	**8.1**	**11.8**	**7.8**	**11.6**	**8.1**	**11.7**
MRI	8.7^††^	14.1^††^	5.9^††^	9.7^††^	8.9	14.8^††^	7.7	12.2^††^	10.3^††^	15.8^††^	8.2^††^	12.8^††^	9.4^††^	13.5^††^	7.5^††^	10.8^††^	8.1^††^	13.3^††^	6.7^††^	10.4^††^	7.3^††^	11.3^††^	5.8^††^	8.6^††^	**8.6^††^**	**13.4^††^**	**6.8^††^**	**10.5^††^**	**8.4**	**13.1**
Mammography	13.8^††^	42.9	11.5^††^	39.0	13.8	44.3	12.5	41.1	13.7^††^	43.4	12.0^††^	41.2	13.0	35.8^††^	13.3	33.0^††^	14.0	41.3	13.3	40.9	13.4	33.0	14.1	33.6	**13.6**	**39.0^††^**	**13.1**	**37.5^††^**	**13.5**	**38.9**
Genetic counseling	5.3^††^	—	3.8^††^	—	5.7^††^	—	3.8^††^	—	7.1^††^	—	4.3^††^	—	7.4^††^	—	3.6^††^	—	6.6^††^	—	4.7^††^	—	8.0^††^	—	5.2^††^	—	**6.9^††^**	—	**4.4^††^**	—	**6.7**	—

**Source:** Truven Health Analytics. Truven Health MarketScan research databases, commercial claims and encounters Medicare supplemental, data year 2009–2014. Ann Arbor, MI: Truven Health Analytics. http://truvenhealth.com/markets/life-sciences/products/data-tools/marketscan-databases

**Abbreviations:** *BRCA* = breast cancer (gene); CPT = Current Procedural Terminology; HCPCS = Healthcare Common Procedure Coding System; ICD-9-CM = *International Classification of Diseases, Ninth Revision, Clinical Modification;* MRI = magnetic resonance imaging; nonmetro = nonmetropolitan.

* Pairwise comparison of rates of preventive services, metropolitan versus nonmetropolitan, for each year and total across study years, by chi-square test.

^†^ Women who had *BRCA* testing for Ashkenazi mutations were excluded from the analyses.

**^§^** Data combined for women aged 18–64 years who had *BRCA* tests, 2009–2014, corrected for the <1% of women who had *BRCA* testing in >1 year.

^¶^ Mastectomy, MRI, and mammography: within 90 days after *BRCA* testing; genetic counseling: within 90 days before and 90 days after *BRCA* testing. Preventive services were defined based on the following billing codes: mastectomy (CPT codes 19303 and 19304 and ICD-9-CM procedure codes 85.33–85.36 and 85.41–85.44), breast MRI (CPT code 77059), and screening and diagnostic mammography (HCPCS codes G0202, G0204, and G0206 and CPT codes 77055, 77056, and 77057).

** Within 1 year after *BRCA* testing.

^††^ p<0.05, chi-square test.

## Discussion

From 2009 to 2014, *BRCA* testing rates among women aged 18–64 years with employer-sponsored health insurance were lower in nonmetropolitan areas than in metropolitan areas. However, *BRCA* testing rates increased more in nonmetropolitan areas than metropolitan areas. Although the absolute differences between metropolitan and nonmetropolitan rates increased over the study period, the relative differences decreased. The lower rates of *BRCA* testing reported in nonmetropolitan areas might reflect differences in access to specialty care providers, including cancer genetic service providers. The lower rate also might reflect general factors such as poverty, lower educational attainment, and lack of health insurance, which have previously been implicated in health care and preventive service access disparities in urban and rural areas (*30*). Although significant differences were found in the sample age distribution between metropolitan and nonmetropolitan areas, adjusting the *BRCA* testing rates by age did not change the general findings or conclusions.

*BRCA* mutations are found in all racial/ethnic groups; however, certain populations, notably Ashkenazi Jews, have both higher frequencies of specific *BRCA* mutations and are concentrated in metropolitan areas, which might therefore differentially affect metropolitan and nonmetropolitan *BRCA* testing rates. In this study, women who received *BRCA* testing for Ashkenazi mutations were excluded to minimize potential bias.

The decreased disparity in testing rates between metropolitan and nonmetropolitan areas from 2009 to 2014 could reflect a decreasing reliance on specialists for genetic testing for *BRCA.* In 2013, the USPSTF recommendation stated that trained health professionals, including primary care providers, could provide genetic counseling about *BRCA* mutation testing to identify women deemed to be at risk for *BRCA* mutations based on family history (*1*). Because small proportions of women who received genetic testing for *BRCA* had claims for genetic counseling throughout the period, both in metropolitan and nonmetropolitan areas, differences in access to genetic counseling services are unlikely to have been an important factor; however, genetic counseling billing codes might not include all instances of counseling services (*31*,*32*).

Finally, the coverage of *BRCA* counseling and testing under the Patient Protection and Affordable Care Act (ACA), when these services are provided in accordance with USPSTF guidelines, might have influenced *BRCA* testing rates both in urban and rural areas during the study period. Although the ACA provision that requires many health plans to promote in-network coverage without cost sharing for certain USPSTF-recommended services became effective in September 2010, a clarification issued in February 2013 might have increased testing, along with other factors discussed (*22*–*24*). This additional guidance specified that *BRCA* testing and counseling, if determined appropriate by a woman’s health care provider, is covered as a preventive service (*10*). A recent study showed that in the 2 years after the 2013 ACA clarification, the percentage of women with no out-of-pocket expenses increased (*33*).

The relative difference in testing rates between metropolitan and nonmetropolitan areas generally decreased over the study period across all age groups but decreased most among women aged 18–34 years and least among women aged 55–64 years. Women aged 18–34 years had the highest relative growth in *BRCA* testing compared with older age groups for both metropolitan and nonmetropolitan areas. Younger persons might adopt the use of newer health care services more quickly, and access to services might present less of a barrier for younger than for older age groups (*34*). The regional analyses demonstrated that national increases in *BRCA* testing can mask important differences in population subgroups. For example, in the West, *BRCA* testing increased more over the study period in metropolitan areas than in nonmetropolitan areas, unlike the other regions examined. This might indicate that the West has unique challenges in reaching nonmetropolitan areas with *BRCA* testing.

Among women with *BRCA* mutations, lower receipt of preventive services such as MRI in nonmetropolitan areas could reflect barriers to access, lower risk for having a *BRCA* mutation among those tested, or a higher proportion of *BRCA* testing performed based on family health history compared with personal history of cancer. The percentage of women who received a mastectomy or an MRI after *BRCA* testing peaked in 2011 both in metropolitan and nonmetropolitan areas; the decreasing percentages observed from 2012 to 2014 could reflect a shift over time toward a higher proportion of *BRCA* testing done based on family health history compared with personal history of cancer (*35*). The percentage of women who received a mastectomy was similar in metropolitan and nonmetropolitan regions within 90 days and 1 year after *BRCA* testing; however, a longer follow-up period might be needed to include prophylactic surgeries in women with *BRCA* mutations who were ascertained based on family health history rather than newly diagnosed breast cancer (*36*).

If *BRCA* testing rates in nonmetropolitan areas were the same as the rates observed in metropolitan areas, approximately 6,600 additional women (39% more) in this sample would have received *BRCA* testing in nonmetropolitan areas from 2009 to 2014. Conversely, if *BRCA* testing rates in metropolitan areas had been the same as the rates observed in nonmetropolitan areas, approximately 41,000 fewer women in this sample (28% fewer) would have received *BRCA* testing in metropolitan areas from 2009 to 2014. If the average metropolitan *BRCA* testing rates from the two regions with the highest rates of testing (Northeast and South) were applied to the two regions with the lowest rates of testing (Midwest and West), 21% more *BRCA* tests would be expected in metropolitan areas and 65% in nonmetropolitan areas.

The highest annual rate of *BRCA* testing reported in this study (332.5 women with any *BRCA* test per 100,000 women aged 44–54 years in 2014) is comparable to the upper-bound estimated prevalence of *BRCA* mutations in the general U.S. population of 200–333 per 100,000 (*37*). However, in Michigan during 2009–2013, <15% of women tested after counseling had a *BRCA* mutation identified (*38*), which is consistent with the predictive value of current risk assessment criteria (*1*). Improved collection of family health history information and assessment of this information using validated risk assessment tools could help improve the identification of women at increased risk for *BRCA* mutations who could benefit from genetic counseling, testing, and preventive interventions. Cascade testing of family members for mutations that have been identified previously in relatives can increase the potential impact of *BRCA* testing. However, few women who have *BRCA* testing are tested for known mutations (4%–5% in this study), which indicates that the frequency of cascade testing is low.

## Limitations

The findings in this report are subject to at least five limitations. First, MarketScan CCAE databases include women who have employer-sponsored insurance but not those who are uninsured, have public insurance, or purchase insurance themselves. These data are not representative of the general U.S. population and might overestimate *BRCA* testing rates. Approximately 60% of working-age U.S. adults have employer-sponsored insurance; this percentage has not changed appreciably in recent years (*39*). In addition, the sample is not necessarily representative of the population with employer-sponsored insurance (*40*); the number of plans and enrollees in the MarketScan CCAE databases varied over the study period. Second, the MarketScan analysis does not include *BRCA* tests that were paid for by the patient or those ordered under CPT codes for laboratory methods and test reporting that are not specific to *BRCA* testing. Claims data also might be subject to recording errors or adjudication errors, and the reliability of the coding of *BRCA* tests might have changed over time. These factors might have affected the completeness of ascertainment of *BRCA* tests, particularly before 2013 (*21*,*41*). Third, the OMB metropolitan-nonmetropolitan classification scheme is an imperfect proxy for rural-urban classification for several reasons. For example, large metropolitan areas might contain rural areas that might be far from an urban center, which might reduce the overall rates of *BRCA* testing observed in metropolitan areas and present an incomplete assessment of *BRCA* testing in nonmetropolitan areas due to misclassification. In addition, residence in a metropolitan or nonmetropolitan area was determined by the primary beneficiary, who might have lived in a different area than the woman who received the test (e.g., young women who were on a parent health insurance plan but did not live with the parent and spouses who were on the same insurance plan but lived in different states). Residence in a metropolitan or nonmetropolitan area might have changed at the time of *BRCA* testing and might also have changed for multiyear enrollees. Among women who had *BRCA* testing, <1% had testing over multiple years, and <1% of those changed residential status. Fourth, the outcomes of *BRCA* tests and whether women met current *BRCA* testing guidelines could not be determined from medical claims data. Therefore, determining whether the lower levels of testing in nonmetropolitan areas represented proportionately lower use of appropriate testing compared to metropolitan areas was not possible. Finally, the billing codes used to identify genetic counseling services were expected to identify only a fraction of women who received genetic counseling because genetic counseling is often billed under evaluation and management codes when attended by a physician (*31*,*32*), and counseling might be provided by clinicians other than genetic counselors. Therefore, these findings underestimate receipt of genetic counseling services. Mammography and MRI percentages are likely overestimates because both screening and diagnostic indications are included (*42*).

## Conclusion

Although *BRCA* testing rates were lower in nonmetropolitan areas, they increased proportionally more than in metropolitan areas. Regional and age differences in *BRCA* testing rates reveal specific population subgroups with greater metropolitan and nonmetropolitan differences. Administrative data used in this report can help public health officials monitor changes over time in the differences in *BRCA* testing rates and receipt of preventive services between metropolitan and nonmetropolitan populations to address differences in *BRCA* testing rates, specifically, and (possibly) genetic services, in general. However, administrative data cannot be used to assess compliance with recommendations for testing and preventive services. To monitor the extent to which *BRCA* testing rates and receipt of preventive interventions after *BRCA* testing adhere to evidence-based guidelines and result in actions likely to prevent death from certain cancers, improved surveillance tools are needed to determine the indications for and results of *BRCA* testing. CDC funds state health departments to promote the application of best practices for evidence-based breast cancer genomics through education, surveillance, and policy activities (*43*). The data sources, tools, and resources developed through these programs can be applied to further examine and address differences in rates of *BRCA* testing and receipt of follow-up preventive services across population subgroups, including metropolitan and nonmetropolitan differences. Programs can build on the recent decrease in geographic disparities in receipt of *BRCA* testing while simultaneously educating the public and health care providers about U.S. Preventive Services Task Force recommendations and other clinical guidelines for *BRCA* testing and counseling.

## References

[1] Moyer VA; US Preventive Services Task Force. Risk assessment, genetic counseling, and genetic testing for *BRCA*-related cancer in women: U.S. Preventive Services Task Force recommendation statement. Ann Intern Med 2014;160:271–81. 24366376 10.7326/M13-2747

[2] Howlader N, Noone AM, Krapcho M, ., eds. SEER cancer statistics review, 1975–2012. Bethesda, MD: National Cancer Institute; 2015. http://seer.cancer.gov/csr/1975_2012

[3] National Comprehensive Cancer Network. National Comprehensive Cancer Network clinical practice guidelines in oncology. Genetic/Familial high risk assessment: breast and ovarian. Version 2.2017. Fort Washington, PA: National Comprehensive Cancer Network, Inc; 2017.

[4] Kim G, Ison G, McKee AE, FDA approval summary: Olaparib monotherapy in patients with deleterious germline *BRCA*-mutated advanced ovarian cancer treated with three or more lines of chemotherapy. Clin Cancer Res 2015;21:4257–61. 10.1158/1078-0432.CCR-15-0887 26187614

[5] Fayanju OM, Hwang ES. Contralateral prophylactic mastectomy: aligning patient preferences and provider Recommendations. JAMA Surg 2017;152:282–3. 10.1001/jamasurg.2016.4750 28002558

[6] US Food and Drug Administration. Niraparib (Zejula). Washington, DC: US Food and Drug Administration; 2017. https://www.fda.gov/Drugs/InformationOnDrugs/ApprovedDrugs/ucm548487.htm

[7] US Food and Drug Administration. FDA grants accelerated approval to new treatment for advanced ovarian cancer. Washington, DC: US Food and Drug Administration; 2017. https://www.fda.gov/newsevents/newsroom/pressannouncements/ucm533873.htm

[8] Robson M, Im SA, Senkus E, Olaparib for metastatic breast cancer in patients with a germline *BRCA* mutation. N Engl J Med 2017;NEJMoa1706450. 10.1056/NEJMoa1706450 28578601

[9] US Department of Health and Human Services. Healthy people 2020. Topics and objectives: genomics. Washington, DC: US Department of Health and Human Services; 2011. Available at https://www.healthypeople.gov/2020/topics-objectives/topic/genomics

[10] US Department of Labor. FAQs about Affordable Care Act Implementation (part XII). Washington, DC: US Department of Labor; 2013. https://www.dol.gov/sites/default/files/ebsa/about-ebsa/our-activities/resource-center/faqs/aca-part-xii.pdf

[11] Hampel H, Bennett RL, Buchanan A, Pearlman R, Wiesner GL; Guideline Development Group, American College of Medical Genetics and Genomics Professional Practice and Guidelines Committee and National Society of Genetic Counselors Practice Guidelines Committee. A practice guideline from the American College of Medical Genetics and Genomics and the National Society of Genetic Counselors: referral indications for cancer predisposition assessment. Genet Med 2015;17:70–87. 10.1038/gim.2014.147 25394175

[12] National Society of Genetic Counselors. Professional status survey: executive summary. Chicago, IL: National Society of Genetic Counselors; 2016. http://www.nsgc.org/p/cm/ld/fid=68

[13] Kinney AY, Steffen LE, Brumbach BH, Randomized noninferiority trial of telephone delivery of *BRCA1*/2 genetic counseling compared with in-person counseling: 1-year follow-up. J Clin Oncol 2016;34:2914–24. 10.1200/JCO.2015.65.9557 27325848 PMC5012661

[14] Senier L, Kearney M, Orne J. Using public-private partnerships to mitigate disparities in access to genetic services: lessons from Wisconsin. Adv Med Sociol 2015;16:269–305. 10.1108/S1057-629020150000016010 27279725 PMC4894330

[15] Hilgart JS, Hayward JA, Coles B, Iredale R. Telegenetics: a systematic review of telemedicine in genetics services. Genet Med 2012;14:765–76. 10.1038/gim.2012.40 22498847

[16] McDonald E, Lamb A, Grillo B, Lucas L, Miesfeldt S. Acceptability of telemedicine and other cancer genetic counseling models of service delivery in geographically remote settings. J Genet Couns 2014;23:221–8. 10.1007/s10897-013-9652-9 24014153

[17] Venne V, Meyer LJ. Genetics and the veterans health administration. Genet Med 2014;16:573–5. 10.1038/gim.2014.7 24625445

[18] Keating NL, Stoeckert KA, Regan MM, DiGianni L, Garber JE. Physicians’ experiences with *BRCA1*/2 testing in community settings. J Clin Oncol 2008;26:5789–96. 10.1200/JCO.2008.17.8053 19001322 PMC2645103

[19] Weitzel JN, Blazer KR, MacDonald DJ, Culver JO, Offit K. Genetics, genomics, and cancer risk assessment: state of the art and future directions in the era of personalized medicine. CA Cancer J Clin 2011;61:327–59. 21858794 10.3322/caac.20128 PMC3346864

[20] HIPAASpace [Internet]. S3818: HCPCS 2017 code. HIPAASpace; 2017. https://www.hipaaspace.com/Medical_Billing/Coding/Healthcare.Common.Procedure.Coding.System/S3818.

[21] Lynch J, Berse B. Methods to identify *BRCA* testing in claims data. Am J Obstet Gynecol 2016;215:133–4. 10.1016/j.ajog.2016.03.049 27083760

[22] Roberts MC, Dusetzina SB. The effect of a celebrity health disclosure on demand for health care: trends in *BRCA* testing and subsequent health services use. J Community Genet 2017;8:141–6. 10.1007/s12687-017-0295-7 28299592 PMC5386917

[23] Desai S, Jena AB. Do celebrity endorsements matter? Observational study of *BRCA* gene testing and mastectomy rates after Angelina Jolie’s New York Times editorial. BMJ 2016;355:i6357. 10.1136/bmj.i6357 PMC5156611 27974323

[24] Myers MF, Chang MH, Jorgensen C, Genetic testing for susceptibility to breast and ovarian cancer: evaluating the impact of a direct-to-consumer marketing campaign on physicians’ knowledge and practices. Genet Med 2006;8:361–70. 10.1097/01.gim.0000223544.68475.6c 16778598

[25] Cragun D, Weidner A, Lewis C, Racial disparities in *BRCA* testing and cancer risk management across a population-based sample of young breast cancer survivors. Cancer 2017;123:2497–505. 10.1002/cncr.30621 28182268 PMC5474124

[26] Cragun D, Bonner D, Kim J, Factors associated with genetic counseling and *BRCA* testing in a population-based sample of young Black women with breast cancer. Breast Cancer Res Treat 2015;151:169–76. 10.1007/s10549-015-3374-7 25868867 PMC4503247

[27] Truven Health Analytics. Truven Health MarketScan research databases, commercial claims and encounters Medicare supplemental, data year 2009–2014. Ann Arbor, MI: Truven Health Analytics. http://truvenhealth.com/markets/life-sciences/products/data-tools/marketscan-databases

[28] US Census Bureau. Geographic terms and concepts—census divisions and census regions. Washington, DC: US Census Bureau; 2010. https://www.census.gov/geo/reference/gtc/gtc_census_divreg.html.

[29] Freeman JL, Klabunde CN, Schussler N, Warren JL, Virnig BA, Cooper GS. Measuring breast, colorectal, and prostate cancer screening with Medicare claims data. Med Care 2002;40(Suppl):IV-36–IV-42. 10.1097/00005650-200208001-00005 12187166

[30] Agency for Healthcare Research and Quality. 2014 National Healthcare Quality and Disparities Report chartbook on rural health care. Pub. No. 15–0007–9-EF. Rockville, MD: Agency for Healthcare Research and Quality; 2015. https://www.ahrq.gov/research/findings/nhqrdr/2014chartbooks/ruralhealth/index.html

[31] Leonhard JR, Munson PJ, Flanagan JD, Analysis of reimbursement of genetic counseling services at a single institution in a state requiring licensure. J Genet Couns 2017;26:852–8. 10.1007/s10897-016-0062-7 28181058

[32] Gustafson SL, Pfeiffer G, Eng C. A large health system’s approach to utilization of the genetic counselor CPT 96040 code. Genet Med 2011;13:1011–4. 10.1097/GIM.0b013e3182296344 21857230

[33] Chen Z, Kolor K, Grosse SD, Trends in utilization and costs of *BRCA* testing among women aged 18–64 years in the United States, 2003–2014. Genet Med 2017. In press. 10.1038/gim.2017.118 PMC8485755 28933789

[34] Heart T, Kalderon E. Older adults: are they ready to adopt health-related ICT? Int J Med Inform 2013;82:e209–31. 10.1016/j.ijmedinf.2011.03.002 21481631

[35] Guo F, Hirth JM, Lin YL, Use of *BRCA* mutation test in the U.S., 2004–2014. Am J Prev Med 2017;52:702–9. 10.1016/j.amepre.2017.01.027 28342662 PMC5370584

[36] Evans DG. Re: Do celebrity endorsements matter? Observational study of *BRCA* gene testing and mastectomy rates after Angelina Jolie’s New York Times editorial. Questionable Questions, Questionable answers. BMJ 2016;355:i6357. 10.1136/bmj.i6357 PMC5156611 27974323

[37] Nelson HD, Pappas M, Zakher B, Mitchell JP, Okinaka-Hu L, Fu R. Risk assessment, genetic counseling, and genetic testing for *BRCA*-related cancer: systematic review to update the U.S. Preventive Services Task Force recommendation. Evidence synthesis no. 101. Pub. No. 12–05164-EF-1. Rockville, MD: Agency for Healthcare Research and Quality; 2013. https://www.uspreventiveservicestaskforce.org/Page/Document/evidence-summary17/brca-related-cancer-risk-assessment-genetic-counseling-and-genetic-testing 24432435

[38] CDC. Cancer and family history: using genomics for prevention. Atlanta, GA: US Department of Health and Human Services, CDC; 2016 https://www.cdc.gov/cdcgrandrounds/pdf/archives/2016/phgr_april_final.pdf

[39] Carman KG, Eibner C, Paddock SM. Trends in health insurance enrollment, 2013–15. Health Aff (Millwood) 2015;34:1044–8. 10.1377/hlthaff.2015.0266 25947173

[40] Aizcorbe A, Liebman E, Pack S, Cutler DM, Chernew ME, Rosen AB. Measuring health care costs of individuals with employer-sponsored health insurance in the U.S.: A comparison of survey and claims data. Stat J IAOS 2012;28:43–51. 26146526 10.3233/SJI-2012-0743 PMC4486327

[41] Wright JD, Chen L, Tergas AI, Underuse of *BRCA* testing in patients with breast and ovarian cancer. Am J Obstet Gynecol 2016;214:761–3. 10.1016/j.ajog.2016.02.011 26875946

[42] Qin X, Tangka FK, Guy GP Jr, Howard DH. Mammography rates after the 2009 revision to the United States Preventive Services Task Force breast cancer screening recommendation. Cancer Causes Control 2017;28:41–8. 10.1007/s10552-016-0835-1 28025762 PMC5865399

[43] Trivers KF, Rodriguez JL, Cox SL, Crane BE, Duquette D. The activities and impact of state programs to address hereditary breast and ovarian cancer, 2011–2014. Healthcare (Basel) 2015;3:948–63. 10.3390/healthcare3040948 27417805 PMC4934623

